# Supplementary material for: GEMTELLIGENCE: Accelerating gemstone classification with deep learning
Source: Commun Eng. 2024 Aug 20;3:110. doi: 10.1038/s44172-024-00252-x (PMC11336078; doi:10.1038/s44172-024-00252-x)
Supplement: Supplementary file 2 — Supplementary Material [file 44172_2024_252_MOESM2_ESM.pdf]

GEMTELLIGENCE: Accelerating Gemstone classification with Deep Learning

Tommaso Bendinelli[1], Luca Biggio[1,2], Daniel Nyfeler[3], Abhigyan Ghosh[3], Peter Tollan[3], Moritz Alexander Kirschmann[1] and Olga Fink[4]

[1]CSEM SA, Alpnach, Switzerland
[2]Data Analytics Lab, ETH, Zürich, Switzerland
[3]Gübelin Gem Lab Ltd, Lucerne, Switzerland
[4]Laboratory for Intelligent Maintenance and Operations Systems, EPFL, Switzerland

Supplementary Notes

Supplementary Note 1: Preliminary experiments

In this Note, we present the performance of GEMTELLIGENCE for OD using FTIR, and for TD using XRF and ICP-MS. Supplementary Fig. 1 demonstrates that FTIR does not provide any additional predictive power when combined with other data sources, which is consistent with laboratory practice, as FTIR does not contribute much to the determination of origin of sapphires. Regarding TD, Supplementary Fig.2 reveals that both ICP-MS and XRF yield sub-optimal results for TD. This is in line with laboratory practice, as elemental analysis methods are not considered meaningful for detecting standard artificial heat treatment of sapphires.

Supplementary Note 2: A closer look at per-class performance

In this Note, we present the per-class performance of GEMTELLIGENCE for both OD and TD. Supplementary Fig. 3 and 4 display the confusion matrices for OD and TD in three operational modes (`None`, `Mode 1`, and `Mode 2`). The OD confusion matrices reveal that GEMTELLIGENCE performs well on stones from Burma, Kashmir, and Sri Lanka while its performance on stones from Madagascar is less accurate. This is expected, as Madagascar stones possess highly diverse features, making consistent OD challenging.[Supp2] In the highest confidence mode, GEMTELLIGENCE achieves nearly zero errors for stones from Burma, Kashmir, and Sri Lanka, while reducing the number of predictions for Madagascar stones; indicating a lower confidence. The TD confusion matrices of GEMTELLIGENCE demonstrate a similar distribution of errors between treated and non-treated stones. Remarkably, by using the high confidence mode, the number of errors can be halved, from 18 in mode `None` to 9 in `Mode 2`, while still allowing the processing of a majority of treated and non-treated stones.

Supplementary Note 3: Comparison with masked model

This Note investigates the impact of employing our masking mechanism (illustrated in Fig. 1 with switch symbols) when utilizing GEMTELLIGENCE on a subset of all the possible data sources. The masking mechanism involves eliminating a portion of the input data sources. The primary objective of this study is to compare the effectiveness of the standard GEMTELLIGENCE version, which is trained on all data sources and then masked, with that of models trained on a single data source. Supplementary Fig. 5 shows that the masked model's performance is comparable to that of the models trained on individual data sources. This finding implies that GEMTELLIGENCE can be efficiently applied in situations where only a subset of data sources is available.

Supplementary Note 4: Comparison with an ensemble model

This Note investigates the impact of processing all data sources end-to-end in the GEMTELLIGENCE architecture, instead of utilizing a set of classifiers trained separately on each data source. To obtain a single prediction, we train a meta-model consisting of a linear layer followed by a softmax operator, which takes the predictions of the single classifiers' (corresponding to the different encoders used in GEMTELLIGENCE) as input and generates the final result. This experiment aims to understand how GEMTELLIGENCE combines information from different data modalities and at which network hierarchy level such fusion occurs. Supplementary Fig. 6 presents a comparison of the GEMTELLIGENCE and ensemble model's performance on the OD and TD tasks. The results show that the two models perform similarly on both tasks, with GEMTELLIGENCE achieving slightly better results on TD. This outcome suggests that while the model effectively combines the representations of various input modalities, the encoders mainly extract coarse high-level features, focusing on more abstract or high-level features. This could be attributed to the model being operated in a low data regime (only a few thousand input samples). Nonetheless, the end-to-end design of GEMTELLIGENCE is expected to offer even more benefits as the training set grows larger,[Supp3] which is anticipated with upcoming data acquisitions. Moreover, using a single end-to-end model offers several practical advantages such as simplicity, maintainability, and robustness, which make it a preferred choice for real-world applications.

Supplementary Note 5: Dataset details and composition

Our training and testing dataset for the main manuscript consists of high-quality metamorphic sapphires. These stones exhibit a rich, saturated, and homogeneous color with high transparency and clarity. They originate from specific geographic locations: Sri Lanka, non-basaltic depositis in Burma/Myanmar and Madagascar, and the Padar area within the Kudi Valley near Sumjam village in the Jammu and Kashmir region of India. We refer to this last location, situated at the northern tip of India between Pakistan and China, as Kashmir. Notably, the Kashmir region produced high-quality blue sapphires during a limited period in the second half of the 19th century. The stones were analyzed between 2013 and 2020 at one three Gübelin Gem laboratories (Lucerne, New York, Hong Kong) or at a mobile laboratories at various gemstone fairs. The ground truth label for OD and TD was determined by expert gemologists by comparing the pattern of gemological properties of the unknown stone with the pattern of properties of stones for which provenance is certain, called reference samples. The entire reference dataset comprises 28,000 stones and provides a comprehensive overview of all the commercially relevant mines of gem-quality corundum worldwide. As explained in the section Training and Testing Datasets, in order to ensure a rigorous analysis, we have restricted the stones in the test set for OD, to stones for which 1) Each measurement is examined independently from others 2) All possible relevant measurements are taken 3) Two expert gemologists independently reach the same conclusion via visual inspection in TD and the results obtained from ICP-MS and visual inspection match in OD.

Supplementary Table 2 shows the number of stones available for each data source for OD and TD. As can be seen, following the stringent requirements for testing stones resulting in the inclusion of only 705 of 1419 stones for OD and 913 of 1452 for TD, encompassing all measurements, in the testing dataset. The biggest factor for difference is due to the fact that data sources are analysed independently only from 2017, while for training we used stones from 2013. Under the protocol introduced in 2017, each data source was analyzed independently, and the resulting sub-conclusions were combined via a weighted mean by different gemologist.

Supplementary Note 6: Detection of non-metamorphic stones

The training set of GEMTELLIGENCE was limited only to metamorphic stones exclusively from Kashmir, Madagascar, Sri Lanka, and Burma. As a result, testing the approach on stones originating from other less-

frequent regions such as Tanzania, Australia or Montana yields unreliable results. However, the limitation imposed by its geographical training constraints can be mitigated by using GEMTELLIGENCE together with any anomaly detection method, either supervised or unsupervised. To show the feasibility of this approach, we have trained a supervised one-classifier anomaly detection method with all the stones in the training set and with additional 70 stones originating from different mining locations than the training stones. The classifier has the same architecture as GEMTELLIGENCE and uses UV and XRF data. The task of the classifier is to determine whether a stone belongs to the training distribution, i.e. its origin is one out of Madagascar, Sri Lanka, Burma or Kashmir or not. Its performance are shown in Supplementary Table 1. As it can be seen, most of in-distribution and out-of-distribution stones are correctly identified, 2455 and 54 respectively. Only a small fraction of stones are either incorrectly classified as coming from the the training classes but instead being from other countries, or vice-versa. The one-classifier can be used as a pre-classifier, where stones identified as "Other" would be redirected to the expert, as shown in Supplementary Fig. 7. Note that when combining the pre-classifier with the threshold in `Mode 1`, only two are then incorrectly classified. In `Mode 2`, no stones are incorrectly classified, demonstrating the effectiveness of the approach overall.

Supplementary Note 7: Examples of the practical application in the gem testing laboratory

To illustrate how GEMTELLIGENCE integrates into routine laboratory testing procedures, we present two examples of blue sapphires received in the lab with unknown origin and treatment history. In the traditional approach, human experts analyze microscopic observations and spectroscopic data independently, reaching three preliminary conclusions that are then combined into a final assessment.

For the first gemstone, microscopic observations were ambiguous between Sri Lanka, Madagascar and Kashmir origins. Banding, which can be indicative for Kashmir, appeared atypical for this origin, with pronounced blue colours and a lack of parquet structures. UV and XRF measurements were also inconclusive when analysed using classical approaches, with both sources showing properties shared by sapphires from all three origins. Consequently, gemologists were unable to confidently determine the gemstone's origin. However, applying GEMTELLIGENCE to the UV, XRF and FTIR datasets yielded a much more definitive result, indicating a strong preference for Kashmir over either Sri Lanka or Madagascar, with a confidence of 93.86%. In order to obtain more concrete additional evidence, advanced trace and minor element analysis was conducted by ICP-MS. This data was subsequently included in the GEMTELLIGENCE assessment, now classifying the stone as Kashmir with an even higher level of confidence (95.43%). This finding was further supported by in-house interpretation of the ICP-MS data, involving an automated comparison of the unknown data with reference material from each potential origin location.

In the second gemstone, the sub-conclusions drawn by human experts did not clearly indicate a single origin: while microscopic indications pointed towards Burma, Madagascar and Sri Lanka, the spectroscopic data suggested Kashmir, Madagascar and Sri Lanka. Trace element concentrations favored Sri Lanka over Burma and Madagascar. Processing UV, FTIR and XRF data through Gemtelligence suggested Sri Lanka with a confidence of 96.47%. Both these examples demonstrate how GEMTELLIGENCE can support gemological experts in the process of origin determination and even eliminate the need of additional expensive measurements.

Supplementary Note 8: Comparison of GEMTELLIGENCE and gemological experts for heat treatment

When determining whether a gemstone has undergone heat treatment or not, gemologists typically focus on well-established characteristic peaks and shapes in the FTIR and UV spectra. For instance, the presence of a peak at 3232 cm^{-1} is considered diagnostic for heat treatment in metamorphic sapphires, often accompanied by an additional peak at 3309 cm^{-1}.[8] GEMTELLIGENCE is a deep neural network without any prior gemological

knowledge hard-coded into its architecture or loss, meaning that the significance of these peaks is not explicitly encoded in the model. Nevertheless, the network's high performance on TD detection suggests that it has learnt strong diagnostic patterns in the spectral data.

To investigate whether these learnt patterns includes domain expert knowledge, we conducted the following experiment. We took spectra of untreated gemstones and edited them by introducing features characteristic of treated stones. Specifically, we focused on the peaks at 3232 and 3309 cm^{-1}, as these are relatively easy to artificially create. We modeled them, as a first approximation, as Gaussian peaks. Supplementary Fig. 8 shows three different examples of spectra before and after the introduction of these peaks. For comparison, Supplementary Fig. 9 presents three spectra from treated stones. We applied this manipulation to all the non-treated stones and measured the *difference* in the assigned probability of heat treatment before and after the augmentation. Our findings reveal a dramatic increase in the probability of a stone being classified as heat-treated, with a median prediction confidence increasing by 96%. This means, for example, that a stone previously classified as untreated (treated) with a 98% (2%) confidence could, after the spectra manipulation, have a 98% (2%) probability of being heat-treated (untreated).

It is worth noting that the position of the peaks plays a crucial role. If instead of 3232 or 3309 cm^{-1} we add peaks at random wavelengths, these will not impact GEMTELLIGENCE, i.e. the prediction will remain similar to the one without the artificial peaks. It's important to remark that while while this approach of synthetically modifying peaks in the FTIR spectra provides insights into GEMTELLIGENCE, it is not comprehensive. Other aspect such global features in the FTIR spectra, multi-modal features of FTIR and UV, different shapes, might have been learned by the network during the optimisation process. Readers interested in exploring additional combinations can investigate further in our released code to test various frequency bands.

Supplementary Tables

	Training origins	Other
Training origins	2455	4
Other	7	54

Supplementary Table 1. Confusion matrix results of the pre-classifier. "Training origins" refers to the stones' origins used for training GEMTELLIGENCE.

		OD	TD
**Available stones**	*UV*	2978	2953
	FTIR	3355	3981
	XRF	4752	5152
	ICP-MS	2809	3071
	All	1419	1452
	Any	5515	5889
**Test stones**	*UV*	705	913
	FTIR	705	913
	XRF	705	913
	ICP-MS	705	913
	All	705	913
	Any	705	913

Supplementary Table 2. Breakdown of the total stones available and the testing subset for task and data source. The term *All* indicates stones with measurements from all data sources (intersection of all the data sources), while *Any* includes stones with measurements from at least one data source (union of the data sources).

Supplementary Figures

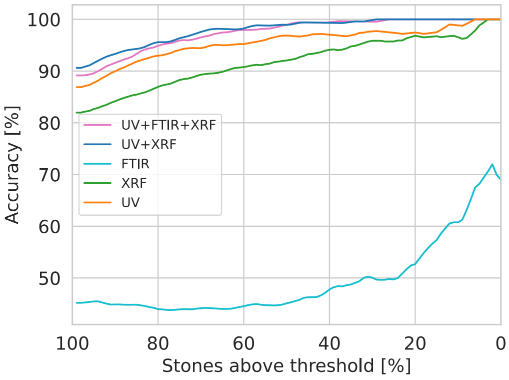

Supplementary Fig. 1. Accuracy (%) vs. stones above the threshold (%) for OD with different data sources provided as input to the model. FTIR performs sub-optimally compared to other data sources. Data sources that are not present in the legend are masked.

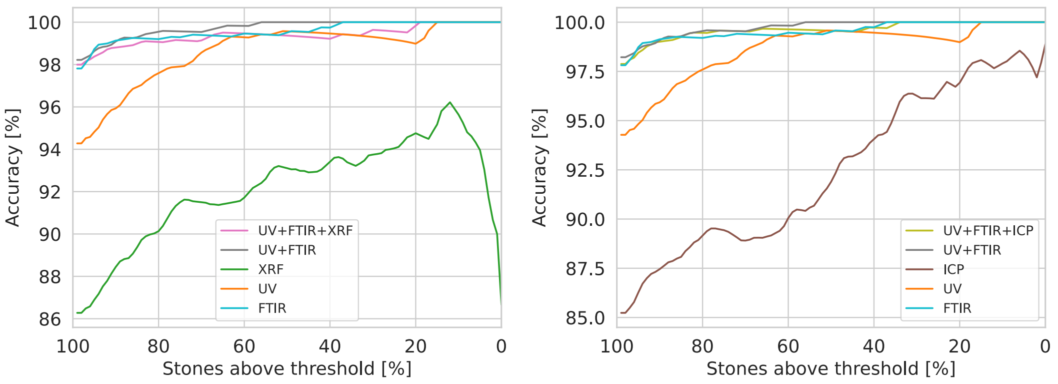

Supplementary Fig. 2. Accuracy [%] vs. stones above the threshold [%] for TD with XRF (Left) and ICP-MS (right). Both XRF and ICP-MS perform sub-optimally compared to other data sources. Data sources that are not present in the legend are masked.

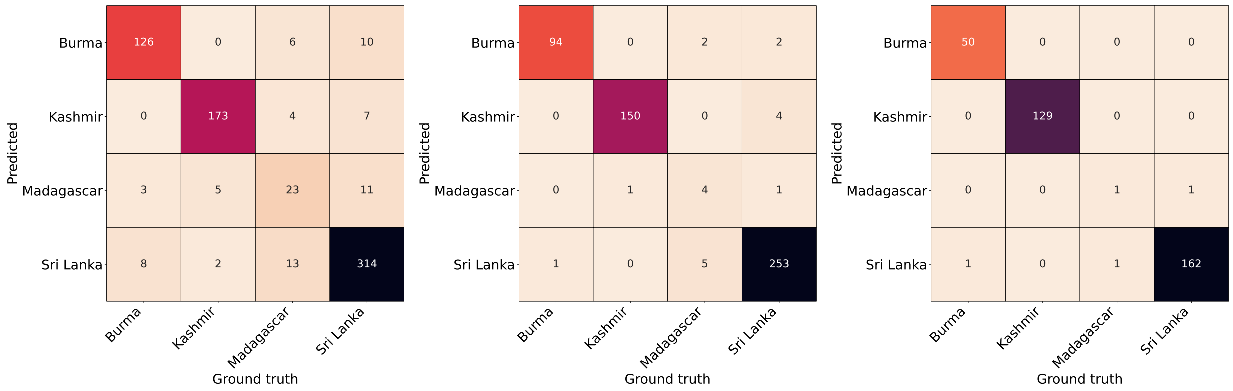

Supplementary Fig. 3. Confusion matrices for OD in the three considered operating modes, namely (Left) `None`, (Middle) `Mode 1`, and (Right) `Mode 2`.

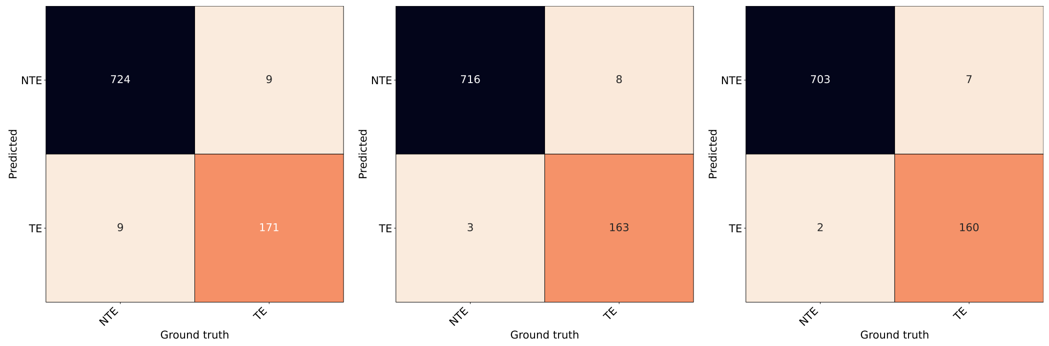

Supplementary Fig. 4. Confusion matrices for TD in the three considered operating modes, namely (Left) `None`, (Middle) `Mode 1`, and (Right) `Mode 2`.

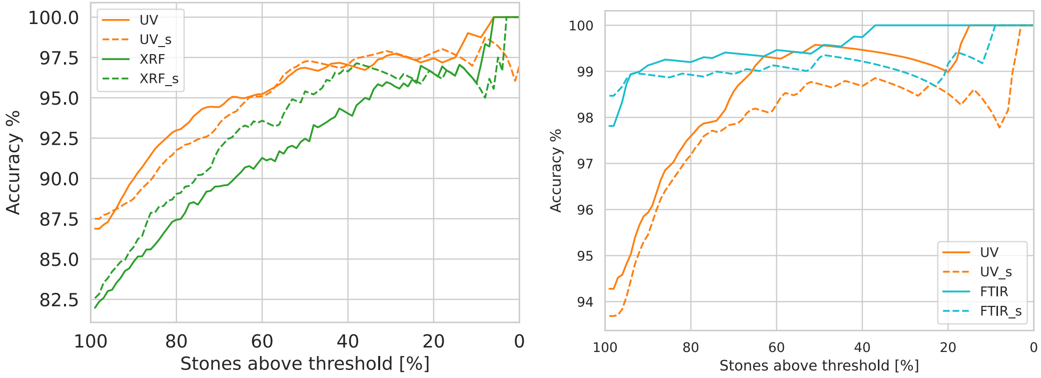

Supplementary Fig. 5. Accuracy [%] vs. stones above the threshold [%] for (Left) OD and (Right) TD. Dashed lines are used to indicate the models trained exclusively on a single data source while full lines indicate GEMTELLIGENCE appropriately masked to hide non-accessible data sources.

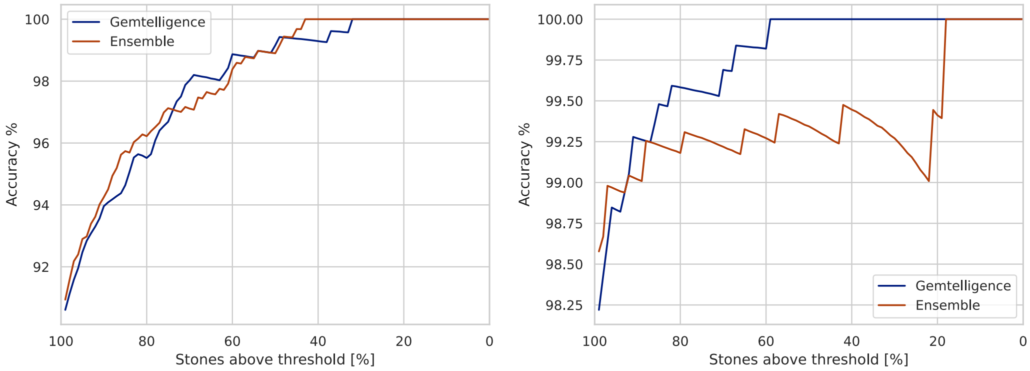

Supplementary Fig. 6. Accuracy [%] vs. stones above the threshold [%] for (Left) OD and (Right) TD. Both panels show a comparison between the ensemble model (orange) and the GEMTELLIGENCE (blue).

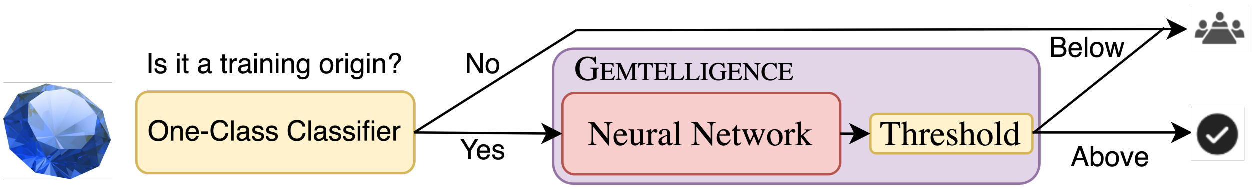

Supplementary Fig. 7. Pipeline when using a one-class pre-classifier. "Training origin" refers to the mining locations of the stones used to train GEMTELLIGENCE, namely Kashmir, Madagascar, Sri Lanka, and Burma.

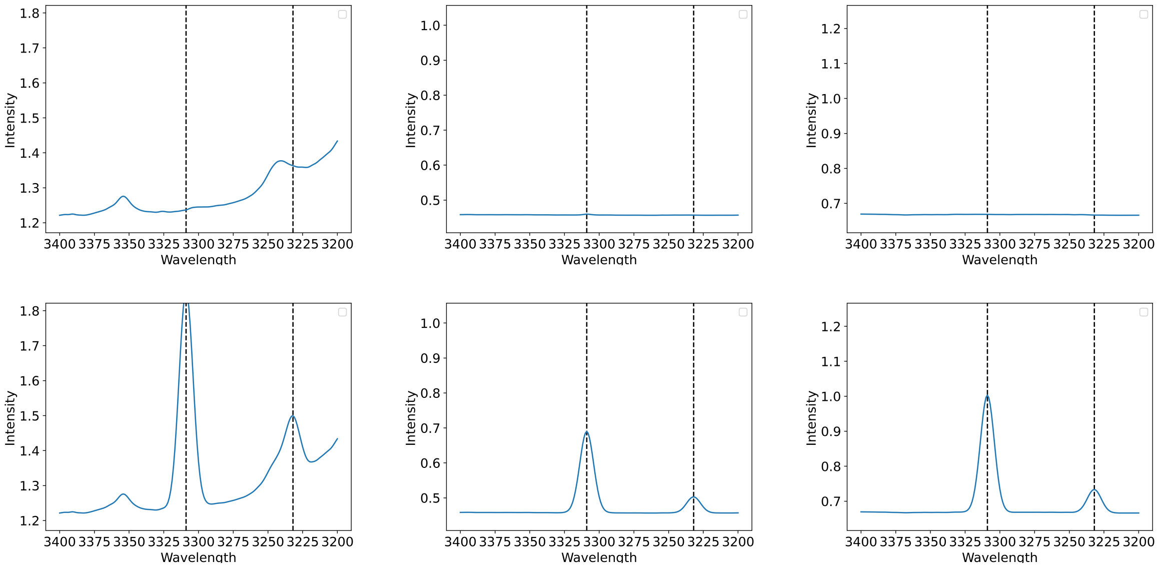

Supplementary Fig. 8. Top row: 3400-3200 cm^{-1} wavelength region in FTIR spectra for untreated sapphires. Bottom row: corresponding augmented spectra. Peaks have a Gaussian shape, with amplitude equal to 50% and 10% of the original values at 3309 and 3232 cm^{-1} respectively and standard deviation of 5 cm^{-1}. For these spectra, the prediction confidence of heat treatment increases by 50%, 98% and 97% respectively. Note that GEMTELLIGENCE receives the entire spectra from 200 cm^{-1} to 7000 cm^{-1}, and the zoomed view is only reported for ease of visualization. Vertical dashed lines denote the 3309 and 3232 cm^{-1} peaks.

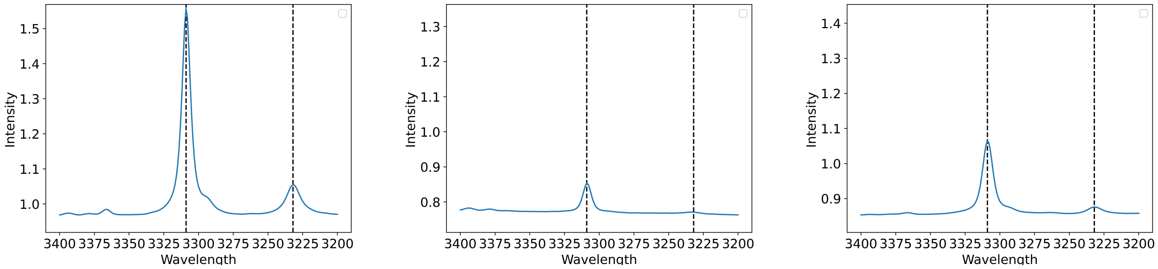

Supplementary Fig. 9. 3400-3200 cm^{-1} wavelength region in FTIR spectra for heat-treated stones that exhibit the characteristic peaks at 3309 cm^{-1} (major) and 3232 cm^{-1} (minor).

Supplementary References

[Supp1] George Forman and Martin Scholz. Apples-to-apples in cross-validation studies: pitfalls in classifier performance measurement. *Acm Sigkdd Explorations Newsletter*, 12(1):49–57, 2010.

[Supp2] Mandy Y Krebs, Matthew F Hardman, David G Pearson, Yan Luo, Andrew J Fagan, and Chiranjeeb Sarkar. An evaluation of the potential for determination of the geographic origin of ruby and sapphire using an expanded trace element suite plus sr–pb isotope compositions. *Minerals*, 10(5):447, 2020.

[Supp3] Andrew Jaegle, Sebastian Borgeaud, Jean-Baptiste Alayrac, Carl Doersch, Catalin Ionescu, David Ding, Skanda Koppula, Daniel Zoran, Andrew Brock, Evan Shelhamer, Olivier Hénaff,

Matthew M. Botvinick, Andrew Zisserman, Oriol Vinyals, and João Carreira. Perceiver io: A general architecture for structured inputs outputs, 2022.

[Supp4] E Billie Hughes and Rosey Perkins. Madagascar sapphire: Low-temperature heat treatment experiments. *Gems Gemol*, 55:184–196, 2019.
